# Linc00473 potentiates cholangiocarcinoma progression by modulation of DDX5 expression via miR-506 regulation

**DOI:** 10.1186/s12935-020-01415-4

**Published:** 2020-07-18

**Authors:** Lining Huang, Xingming Jiang, Zhenglong Li, Jinglin Li, Xuan Lin, Zengtao Hu, Yunfu Cui

**Affiliations:** Department of General Surgery, The 2nd Affiliated Hospital of Harbin Medical University, 246 Xuefu-ro, Harbin, 150086 People’s Republic of China

**Keywords:** lncRNA, linc00473, miR-506, DDX5, Cholangiocarcinoma

## Abstract

**Background:**

Cholangiocarcinoma (CCA) is a mortal cancer with high mortality, whereas the function and mechanism of occurrence and progression of CCA are still mysterious. Long non-coding RNAs (lncRNAs) could function as important regulators in carcinogenesis and cancer progression. Growing evidences have indicated that the novel lncRNA linc00473 plays an important role in cancer progression and metastasis. However, its function and molecular mechanism in CCA remain unknown.

**Methods:**

The linc00473 expression in CCA tissues and cell lines was analyzed using qRT-PCR. Gain- and loss-of-function experiments were conducted to investigate the biological functions of linc00473 both in vitro and in vivo. Insights into the underlying mechanisms of competitive endogenous RNAs (ceRNAs) were determined by bioinformatics analysis, dual-luciferase reporter assays, qRT-PCR arrays, RNA immunoprecipitation (RIP) and rescue experiments.

**Results:**

Linc00473 was highly expressed in CCA tissues and cell lines. Linc00473 knockdown inhibited CCA growth and metastasis. Furthermore, linc00473 acted as miR-506 sponge and regulated its target gene DDX5 expression. Rescue assays verified that linc00473 modulated the tumorigenesis of CCA by regulating miR-506.

**Conclusions:**

The data indicated that linc00473 played an oncogenic role in CCA growth and metastasis, and could serve as a novel molecular target for treating CCA.

## Background

Cholangiocarcinoma (CCA) is an epithelial cell malignancy arising in the region between the intrahepatic bile ducts and the ampulla of Vater at the distal end of the common bile duct. Based on anatomical location, CCA can be divided into intrahepatic cholangiocarcinoma and extrahepatic cholangiocarcinoma [1]. In past decades, the number of CCA patients has been remarkably increasing worldwide, especially in East Asia [2]. Surgical resection is the preferred treatment for all CCA subtypes, but most cases are already in the advanced stage when diagnosed [2]. Patients with the inoperable disease generally receive chemotherapy regimens, in which gemcitabine and cisplatin are the most common used. However, along with poor prognosis, conclusive evidence for efficacy is lacking [3]. Therefore, it is urgent to uncover the pathogenesis of CCA progression and to develop corresponding therapeutic strategies.

Long non-coding RNAs (lncRNAs), with no protein-coding capacity, is a class of transcripts with lengths greater than 200 nucleotides [4]. Increasing evidence shows that lncRNAs are important regulatory factors of gene expression and involved in various physiological and pathological processes, including the occurrence and development of tumors [5]. For example, lncRNA ZFAS1 displays oncogenic properties and regulates important processes associated with EMT in head and neck squamous cell carcinoma [6], lncRNA PVT1 acts as an oncogenic gene and plays an important role in radio sensitivity in malignant nasopharyngeal carcinoma via activating the KAT2A acetyltransferase and stabilizing HIF-1α [7]. lncRNA DANCR regulates proliferation and migration by epigenetically silencing FBP1 in tumorigenesis of CCA [8]. Thus, mounting evidence illuminates that the exploration of molecular alteration in CCA would help improve our understanding of the progression and find out potential therapeutic targets.

Linc00473, located on 6p27, is a novel carcinogenic lncRNA [9]. It has been verified that linc00473 is an oncogene in gastric cancer [10], cervical cancer [11], hepatocellular carcinoma [12] and so on. However, the function of linc00473 in CCA is still undiscovered. Thus, our group identified linc00473, which had not been reported in previous studies, and investigated its roles in proliferation, migration and invasion in vitro. In vivo, tumorigenicity was also determined in nude mice model. Additionally, the regulatory mechanism by which linc00473 interacted with miRNA in regulating CCA metastasis was also analyzed.

## Materials and methods

### Microarray and TCGA dataset analysis

The gene expression profiles of CCA that was downloaded from The Cancer Genome Atlas (TCGA) data portal (https://tcga.xenahubs.net/download/TCGA.CHOL.sampleMap/HiSeqV2.gz) were a Level 3 gene expression profile (level 3 data), and this gene expression profile was measured by the University of North Carolina TCGA genome characterization center. Package limma of the R statistical software was used to perform data analysis. The Fold change > 2 and FDR < 0.05 were set as the cut-offs to screen for differentially expressed genes.

### Patient samples and cell lines

All of the human samples were obtained with informed consent from patients with CCA. A total of 60 CCA samples in this study were collected from The 2nd Affiliated Hospital of Harbin Medical University (Heilongjiang Province, China). There were no patients who had received preoperative chemotherapy before this study. This research was approved by the Institute’s Research Ethics Committee of The 2nd Affiliated Hospital of Harbin Medical University and conducted in accordance with the ethical guidelines of the World Medical Association Declaration of Helsinki. All written informed consents had been collected from each patient before surgery.

HCCC-9810, CCLP1 and RBE cells were obtained from the Cell Bank of Type Culture of Chinese Academy of Sciences (Shanghai, People’s Republic of China). The other CCA cells including QBC939, HuCCT1, KMBC and human intrahepatic biliary epithelial cells (HIBEC) were preserved in our laboratory. These cells were cultured in RPMI-1640 (Gibco, Grand Island, NY, USA) or DMEM (Gibco, Grand Island, NY, USA) containing 10% fetal bovine serum (Invitrogen Life Technologies, Carlsbad, CA, USA) in a humidified atmosphere at 37 °C and 5% CO_2_.

### Cell proliferation assays

CCLP1 and HCCC-9810 cells were seeded into 96-well plates at a density of 10 × 10^4^ cells/well 48 h after transfection. Cell viability was evaluated by cell counting kit-8 (CCK-8, Beyotime, Beijing, China) at 24 h, 48 h, 72 h and 96 h. OD value was quantified by a microplate reader at 450 nm.

CCLP1 and HCCC-9810 cells were seeded in 6-well plates at the density of 500 cells/well 48 h after transfection and then cultured at 37 °C in a 5% CO_2_ humidified atmosphere. The medium was changed every 4 days. After 14-days’ culture, the medium was removed and cells were washed twice with PBS. Then cells were fixed in methanol for 20 min and stained with 0.1% crystal violet (Beyotime, Beijing, China) for 30 min at room temperature, washed again and photographed.

### Cell transfection

Two siRNA targeting linc00473 (si-linc00473-1 and si-linc00473-2) and scrambled negative control (si-NC) were synthesized by RiboBio (Guangzhou, China). The sequences of si-linc00473 were as follows (5′-3′): si-linc00473-1: GCGCCGGGAGAUGCAUCACGAUGAA; si-linc00473-2: CCCUGUCUGCAAAGAUCCAGUUUAA. miR-506 mimics/inhibitor was purchased from GenePharma (Shanghai, China). CCLP1 and HCCC-9810 cells were transfected with 100 nM siRNA using LipofectamineTM3000 (Thermo Fisher Scientific, USA) according to the manufacturer’s instruction. The mRNA expression level of LINC00473 was detected by qRT-PCR. The overexpression experiments were performed as previously described [13].

### qRT-PCR analysis and subcellular fractionation address

Total RNA was isolated from the cell lines using Ttizol reagent (Thermo Fisher Scientific, Waltham, MA, USA) according to the manufacturer’s instruction. One microgram of total RNA was reversely transcribed into first-strand cDNA according to the protocol of Transcriptor First Strand cDNA Synthesis Kit (Roche, Germany) [14], PCR was carried out using FastStart Universal SYBR Green Master Kit (Roche, Germany) according to the instruction in BIO-RAD C1000 Thermal Cycler. All special primers are listed in Additional file 1. The PARIS Kit (Life Technologies, Carlsbad, CA, USA) was used to separate nuclear and cytosolic fractions following the manufacturer’s instruction. Each sample was analyzed at least in triplicate.

### Wound-healing assay and transwell assay

CCLP1 and HCCC-9810 were plated in each well of the 12-well plates and incubated to form 80–90% confluence. Then a scratch was performed with pipette tips. Fresh serum-free medium was changed. The wound closing procedure was observed for 24 h, and images were photographed.

Transwell chambers with 8 μm pores (Costar, Corning, NY, USA) were used to perform the invasion assay. Matrigel (BD Biosciences, New Jersey, USA) was coated on the top side of the insert membrane. The upper chamber was added with 200 μl serum-free medium and seeded 5 × 10^4^ cells while the lower chamber was placed with 600 μl medium with 5% FBS. The chambers were maintained at 37 °C, 5% CO_2_ for 24 h. After that, the cells on the top side of the insert membrane were removed by cotton swabs. The inserts were then fixed in methanol for 20 min and stained with 1% crystal violet for 30 min. The cells on the bottom of the membrane were calculated under a microscope and photographed. All experiments were performed in triplicate.

### Western blot assay

Whole-cell lysate preparation and western blot analysis were performed as previously described [15]. An equal amount of protein from each condition was subjected to electrophoresis on 10% SDS-PAGE and subsequently transferred to polyvinylidene difluoride membrane, which was then blocked with 5% BSA in TBST (TBS containing 0.1% Tween-20) at room temperature for one hour. Incubation was conducted with primary antibodies at 4 °C overnight followed by secondary antibodies at room temperature for one hour. The membranes were washed 3 times with washing buffer (PBS containing 0.1% Tween) for 10 min after each incubation. Images were then captured using Densitometry (Quantity One software; Bio-Rad). Anti-DDX5 (the DEAD box protein 5) were obtained from CST (1:1000, Danvers, USA). Anti-GAPDH were obtained from Beyotime (1:1000, Beijing, China).

### Dual-luciferase reporter assay

The sequences containing the wild-type (WT) or mutated (MUT) region DDX5 and linc00473 were synthesized by GenePharma (Shanghai, China) and inserted into a pmirGLO-Report luciferase vector. For the luciferase reporter assay, miR-506 mimics and the respective reporter plasmids were transfected into cells using Lipofectamine 3000 according to the manufacturer’s protocol. After 24 h, the Renilla and Firefly luciferase activities were determined using the Dual-Luciferase Reporter Assay System (Promega) according to the manufacturer’s instructions.

### RNA-binding protein immunoprecipitation (RIP) assay

miR-506 mimics or miRNA was transfected into HCCC-9810 and CCLP1 cells. And the cells were lysed and collected in a RIP lysis buffer kit (Millipore, USA). Human anti-Ago2 (Millipore, USA) or mouse anti-IgG (Millipore, USA) were then conjugated with RNAs magnetic beads. Then the expression levels of purified RNA were determined by qRT-PCR.

### The immunohistochemistry (IHC) assay

IHC analysis was performed as previously described [16]. For this staining, after heat-induced epitope retrieval, paraffin-embedded sections were incubated with 3% H_2_O_2_, and blocked for another 60 min with 3% normal serum buffer. Sections were incubated with primary antibodies overnight at 4 °C. Elite ABC Staining Kit and DAB Peroxidase Substrate Kit (Vector Laboratories, Inc., Burlingame, CA) were used to visualize the staining according to the manufacturer’s instructions. Primary antibodies used are listed below: DDX5 (1:200, CST, Danvers, USA).

### Xenograft mice model

To evaluate the growth potential of CCA cells in vivo, 2 × 10^6^ stable linc00473-overexpressed CCLP1 cells were re-suspended in 10 μL of DMEM medium and then drawn into a 20 μL Hamilton syringe with a 30-gauge needle and injected subcutaneously into the BALB/C nude mice (each group five mice, 6 weeks). Xenograft was measured every 3 days and tumor volume was calculated. All mice were sacrificed at the end of 21 days and the tumor weight was measured. All animal experiments were approved by the Animal Care and Use Committee of The 2nd Affiliated Hospital of Harbin Medical University, all animal experiments were con-ducted according to the Principles of Laboratory Animal Care (National Society for Medical Research). All the experimental procedures were in accordance with the Declaration of Helsinki.

### Statistical analysis

The data were analyzed applying Statistical Program for Social Sciences 19.0 software (SPSS, Chicago, IL, USA) and GraphPad Prism 5.0 (GraphPad Software, LaJolla, CA, USA). Data were presented as mean ± SD and comparisons were calculated by Student’s *t* test (two-sided, unpaired). Pearson’s rank correlation coefficients were used to calculate correlations between the mRNA levels. The Kaplan–Meier method was used to plot survival curves. All experiments were repeated at least three times. *p* < 0.05 was considered to indicate a statistically significant difference.

## Results

### Linc00473 was up-regulated in cholangiocarcinoma tissues and cells

**Fig. 1:**
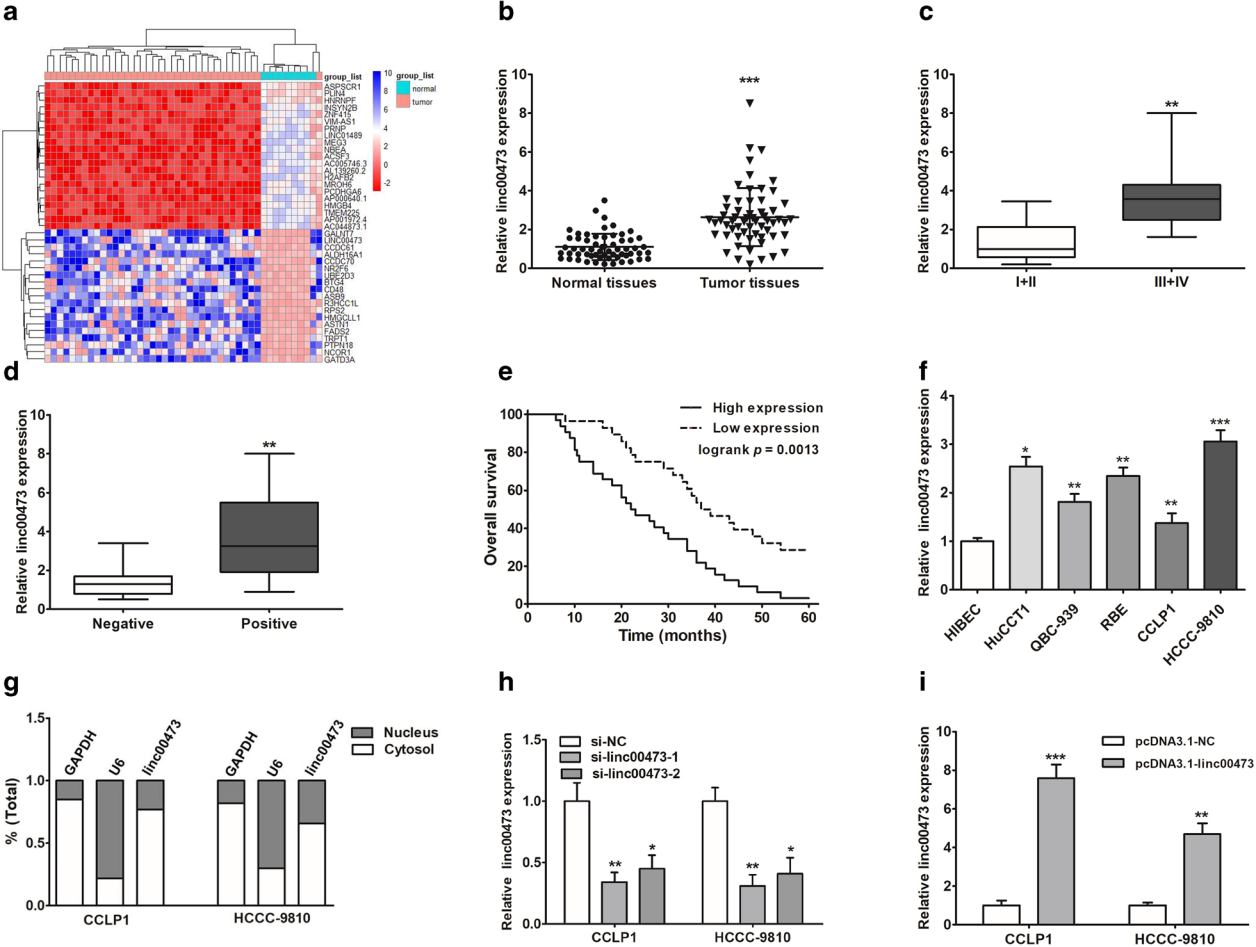
Linc00473 expression level was clearly up-regulated in CCA tissues and cells. **a** Data mining of differentially expressed lncRNAs expression in TCGA dataset. **b** Linc00473 expression was measured by qRT-PCR in CCA tissues and matched adjacent normal tissues (Normal tissues). **c**, **d** The relative linc00473 up-regulation was significantly associated with advanced TNM stage and lymph node invasion. **e** The Correlation between linc00473 expression and the overall survival of CCA patients. **f** Linc00473 expression was up-regulated in five CCA cell lines compare with HIBEC. **g** The linc00473 expression was measured in the nucleus and cytoplasm of CCA cells (CCLP1, HCCC-9810). U6 (nuclear retained) and GAPDH (exported to cytoplasm) were served as controls. **h** CCLP1 and HCCC-9810 cells were transfected with si-NC, si-linc00473 for 24 h, linc00473 expression was using qRT-PCR. **i** Linc00473 expression was significantly increased after transfected with pcDNA3.1-NC/pcDNA3.1-linc00473. The error bars indicate the mean ± SD, and each experiment was repeated at least three times. *p < 0.05, **p < 0.01, ***p < 0.001

To identify differentially expressed lncRNAs in CCA, our group downloaded and analyzed cholangiocarcinoma RNA sequencing (RNA-seq) datasets from TCGA. A total 75 upregulated lncRNAs and 47 downregulated lncRNAs were screened, and the top 20 lncRNAs of significant changes (upregulated and downregulated) were listed in Fig. 1a. Among these lncRNAs, linc00473 was upregulated, and was likely to be of greater utility as diagnostic and prognostic markers. To validate this hypothesis, we determined the expression of linc00473 in CCA tissue samples and their paired nontumor bile duct tissue samples. As shown in Fig. 1b, the content of linc00473 in CCA tissues was significantly higher than that in paired nontumor bile duct tissue samples. To evaluate the correlation between linc00473 expression and the clinical characteristics of the patients with CCA, patients were categorized into low expression (n = 28) group and high expression (n = 32) group according to the median expression level of linc00473. The data showed that the linc00473 expression was correlated with tumor-node-metastasis (TNM) stage, tumor size and lymph node invasion (Table 1). The elevated expression of linc00473 was closely related to the progress of TNM staging (Fig. 1c) and lymph node dilatation (Fig. 1d) in patients with CCA. These results indicated that linc00473 may participate in CCA tumorigenesis. Kaplan–Meier analysis revealed that higher expression of linc00473 was positively linked to a lower overall survival rate (Fig. 1e). Univariate and multivariate analyses were implemented to evaluate prognostic risk factors of CCA patients. The results documented that high linc00473 expression was an independent predictor of poor survival (Table 2). Similarly, the expression of linc00473 was also increased in CCA cell lines (QBC-939, CCLP1, RBE, HuCCT1, and HCCC-9810) when compared with that in the normal bile cell line HIBEC (Fig. 1f). The analysis of the subcellular distribution of linc00473 demonstrated that both CCLP1 and HCCC09810 exhibited higher expression of linc00473 in the cytoplasm than in the nucleus, which showed that linc00473 was distributed in the cytoplasm (Fig. 1g). Among these cell lines, HCCC-9810 had the highest expression of linc00473, and CCLP1 was the lowest one, and these two cell lines were chosen for further experiments. In addition, an efficient down-expression/over-expression of linc00473 in CCLP1 and HCCC-9810 cells were confirmed by qRT-PCR. Linc00473 expression after being transfected with siRNA-linc00473 was markedly decreased than the control group (Fig. 1h), and linc00473 expression after being transfected with pcDNA3.1-linc00473 was significantly higher than the control group (Fig. 1i).

**Table 1:** Association between linc00473 expression and clinicopathological characteristics of CCA patients

	No. of patients (n)	linc00473 expression		*p*-value
		High (n)	Low (n)	
Gender				
Male	26	15	11	0.554
Female	34	17	17	
Age in years				
< 60	19	12	7	0.299
≥ 60	41	20	21	
Tumor location				
Intrahepatic	16	11	5	0.149
Extrahepatic	44	21	23	
Tumor size				
< 3 cm	35	10	25	0.001
≥ 3 cm	25	18	7	
Lymph node invasion				
Positive	38	11	27	0.006
Negative	22	17	5	
TNM stage				
I–II	27	7	20	0.001
III–IV	33	25	8	
Differentiation grade				
Well/moderately	25	13	12	0.943
Poorly/undifferentiated	35	18	16	
HBV infection				
Positive	18	10	8	0.621
Negative	42	22	20	
Serum CEA level (ng/ml)				
> 5	38	22	16	0.352
≤ 5	22	10	12	
Serum CA19-9 level (µ/ml)				
> 37	39	24	15	0.083
≤ 37	21	8	13	

*TNM stage* Tumor-Node-Metastasis stage, *CEA* carcino embryonie antigen, *CA19*-*9* carbohydrate antigen 19-9, *HBV* Hepatitis B virus

**Table 2:** Univariate and multivariate analyses for overall survival of CCA patients

Variables	Univariate analysis			Multivariate analysis		
	HR	95% CI	*p*-value	HR	95% CI	*p*-value
Age (years) < 60 vs. ≥ 60	0.647	0.371–.127	0.124			
Gender Male vs. female	1.500	0.831–2.707	0.178			
Tumor location Extrahepatic vs. intrahepatic	1.118	0.619–2.021	0.712			
Differentiation grade Poor/undifferentiated vs. well/moderate	0.947	0.471–1.904	0.878			
HBV infection Positive vs. negative	0.740	0.320–1.711	0.481			
Serum CEA level (ng/ml) ≤ 5 vs. > 5	1.200	0.568–2.534	0.633			
Serum CA19-9 level (µ/ml) ≤ 37 vs. > 37	1.394	0.645–3.312	0.398			
Tumor size (cm) < 3 vs*. *≥ 3	0.898	0.514–1.571	0.707			
TNM stage I–II vs. III–IV	1.741	1.084–2.686	0.037	1.463	0.958–1.832	0.071
Lymph node invasion Positive vs. negative	1.543	1297–2.211	0.049	2.485	0.914–3.295	0.563
linc000473 expression High vs. low	2.365	1.340–4.173	0.013	2.352	1.302–4.876	0.001

### Knockdown of linc00473 could inhibit growth, invasion, and migration abilities of CCA cells

**Fig. 2:**
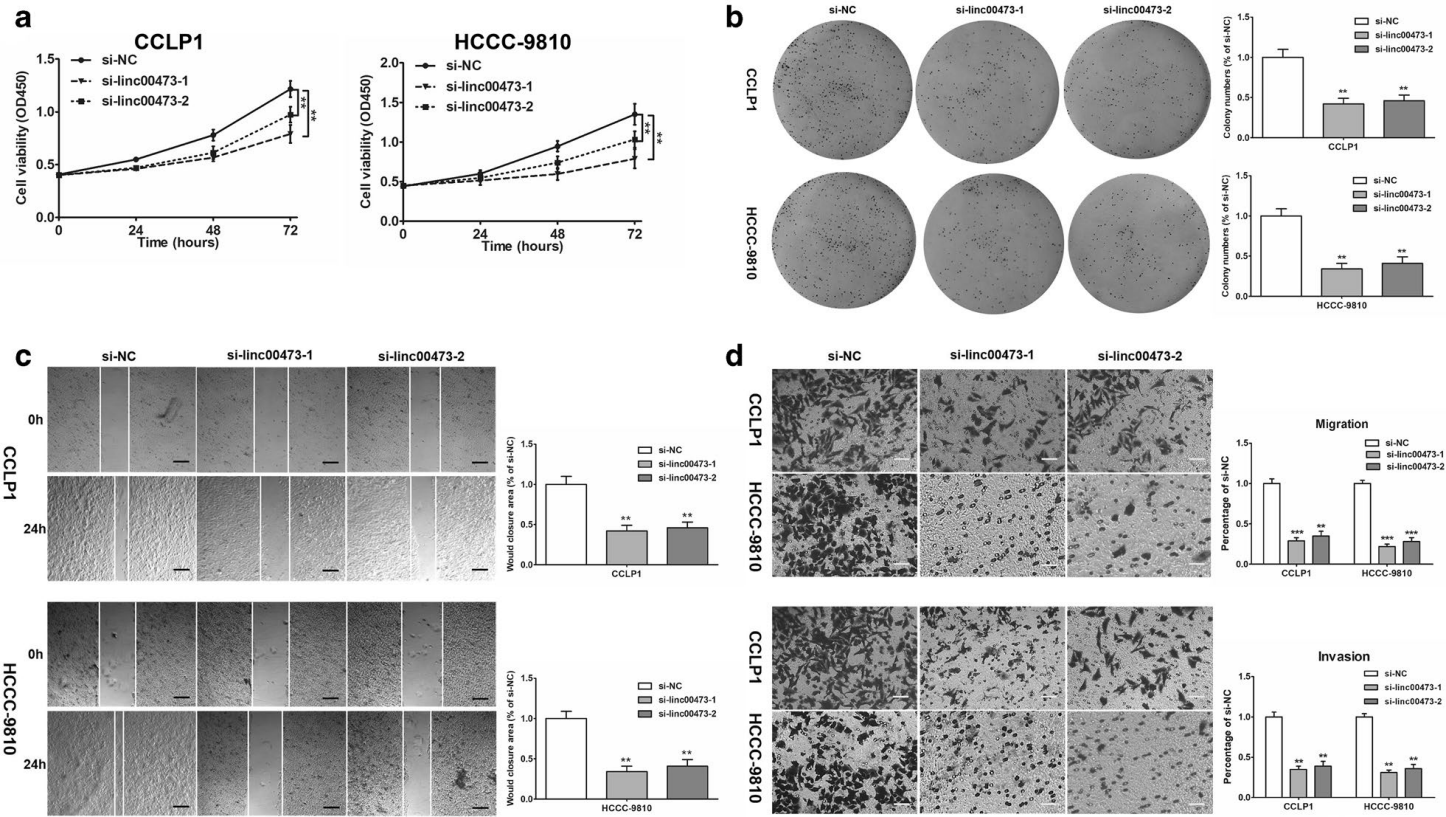
Knockdown of linc00473 inhibited cell proliferation, migration and invasion. **a** The effect of linc00473 knockdown on cell growth of CCLP1 and HCCC-9810 cells detected by the CCK-8 assay. **b** The colony-forming ability of CCLP-1 and HCCC-9810 cells was tested after transfection, and the results demonstrated that silencing linc00473 inhibited colony formation. **c** Silencing linc00473 attenuated wound closure corroborated in CCLP1 and HCCC-9810. **d** The invasive and migration capacities were detected in CCLP1 and HCCC-9810 cells transfected with si-linc00473 or si-NC using transwell assays. The error bars indicate the mean ± SD, and each experiment was repeated at least three times. **p < 0.01, ***p < 0.001

CCK-8 assay revealed that cell proliferation was inhibited in HCCC-9810 and CCLP1 with si-linc00473-1 and si-linc00473-2 transfection compared with that in negative control (si-NC) (Fig. 2a). Consistently, transfection with si-linc00473-1 and si-linc00473-2 significantly suppressed the growth of CCA cells (Fig. 2b). Additionally, wound healing and Transwell assays were inducted to explore the potential impact of linc00473 on migration and invasion in CCA cells. Knockdown of linc00473 with either of the two siRNAs remarkably impaired about half of the wound closure potential (Fig. 2c). Similarly, in Transwell assays, the number of invading cells in the si-linc00473 group was less than that it in the si-NC group (Fig. 2d). The above results indicated that that knockdown of linc00473 could inhibit CCA growth, invasion, and migration abilities of CCA cells.

### Up-regulation of linc00473 promoted CCA cell proliferation, growth and invasion potentials

**Fig. 3:**
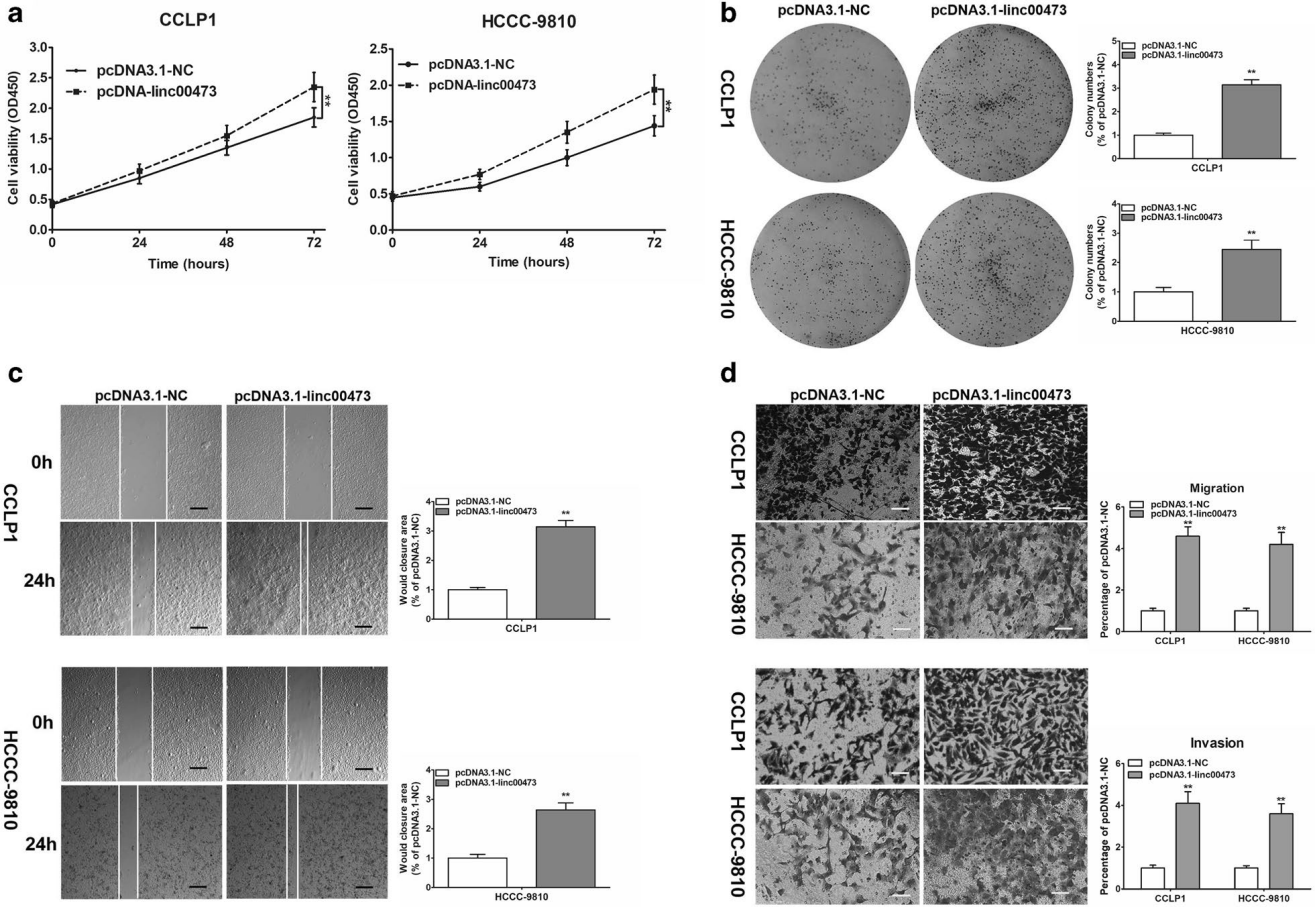
Up-regulation of linc00473 promoted cell proliferation, migration and invasion. **a**, **b** Cell proliferation of CCLP1 after pcDNA3.1 or pcDNA3.1-linc00473 transfection was determined by CCK-8 assay and colony formation assay. **c**, **d** Would-healing assays and transwell assays showed that CCLP1 and HCCC-9801 cells transfected with pcDNA3.1-linc00473 exhibited stronger migration and invasion ability than those transfected with pcDNA3.1-NC. The error bars indicate the mean ± SD, and each experiment was repeated at least three times. **p < 0.01, ***p < 0.001

The cell proliferation and growth potentials were markedly promoted in CCLP1 and HCCC-9810 after being constructed with a linc00473-overexpressing vector (pcDNA3.1-linc00473) (Fig. 3a, b). Also, wound healing and transwell assay showed that the up-regulation of linc00473 enhanced the capacity for migration and invasion in CCA cells (Fig. 3c, d). Collectively, these data suggested that overexpression of linc00473 markedly increased the viability and the migratory and invasive abilities of CCA cells.

### Linc00473 silenced miR-506 expression in CCA cells

**Fig. 4:**
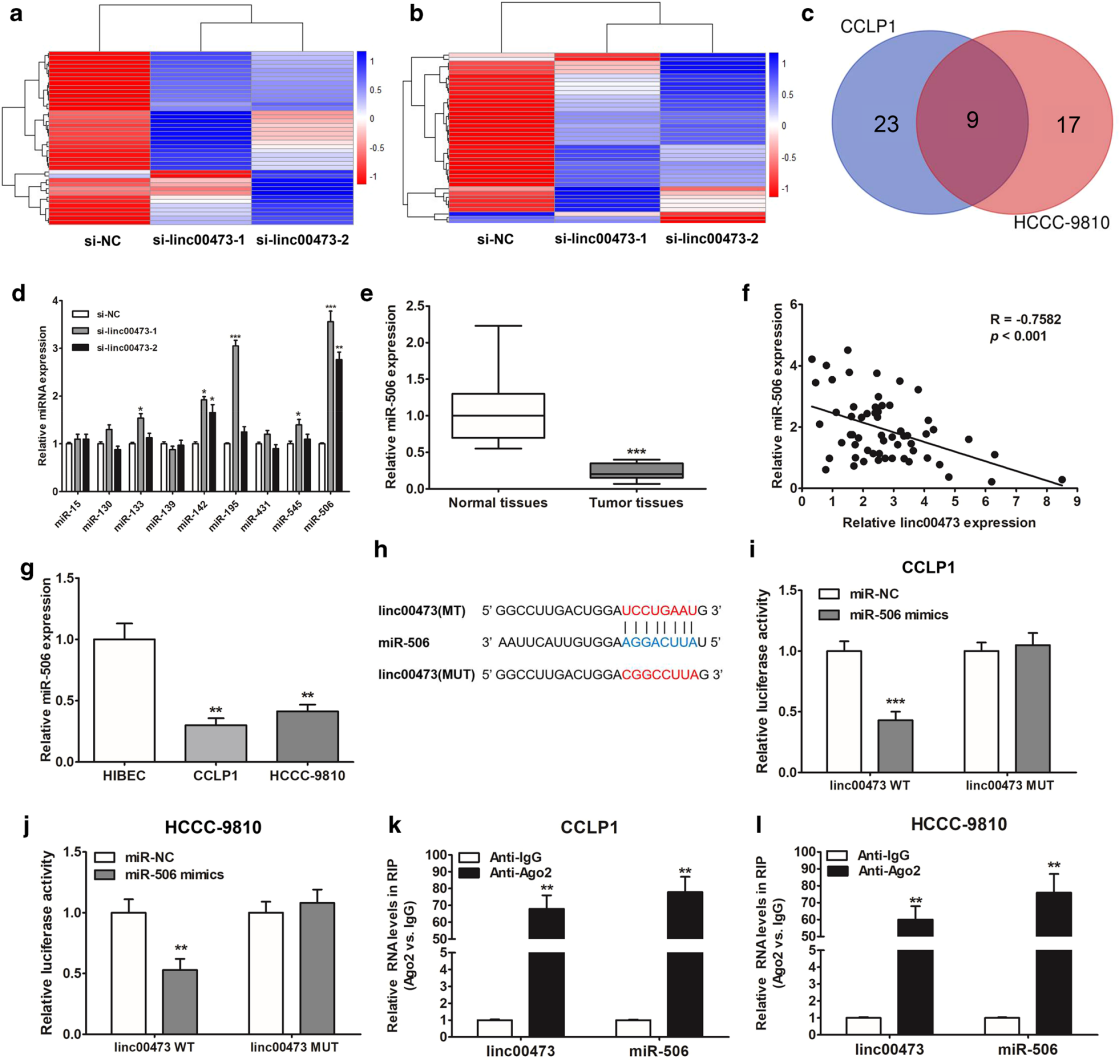
Linc00473 acted as miR-506 sponge in CCA cells. **a**, **b** Heatmaps were drawn to show the differentially expressed miRNAs in CCLP1 and HCCC-9810 cells with depletion of linc00473. **c** Venn diagram shows the overlap between the up-regulated miRNAs in CCLP1 and the up-regulated ones in HCCC-9810. **d** CCLP1 cells were transfected with si-NC, si-linc00473-1 or si-linc00473-2 for 24 h, expression of miRNAs was detected. **e** The differential expression of miR-506 in CCA tissues and adjacent normal bile duct tissues was analyzed by qRT-PCR. **f** Pearson’s correlation curve revealed the negative relevance between linc00473 and miR-506 expression. **g** The miR-506 expression level was investigated in CCA cells (CCLP1, HCCC-9810) and HIBEC. **h** Sequence alignment of miR-506 with the putative binding sites within the wild-type regions of linc00473. **i**, **j** CCLP1 and HCCC-9810 cells were co-transfected with miR-506 mimics and linc00473 WT vector linc00473 MUT vector for 48 h, the luciferase activity was measured. **k**, **l** The expression of linc00473 and miR-506 in the Ago2-containing beads in CCLP1 and HCCC-9810, compared with the beads harboring control IgG. The error bars indicate the mean ± SD, and each experiment was repeated at least three times. **p < 0.01, ***p < 0 .001

To further investigate the mechanism by which linc00473 regulated the proliferation and invasion, we performed global miRNA profiling of CCLP1 and HCCC-9810 cells depleted of linc00473. Expression of a large number of miRNAs was upregulated in cells with knockdown of linc00473 (Fig. 4a, b), we selected miRNAs that were both up-regulated in cells with transfection of si-linc00473-1 and si-linc00473-2, and screened the selected miRNAs for potential binding sites to linc00473 using the biological information database (miRcode, starBase and DIANA) to predict the miRNAs that could bind to linc00473. The nine miRNAs (miR-15, miR-130, miR-139, miR-142, miR-195, miR-431, miR-545, and miR-506) were screened in both CCLP1 and HCCC-9810 cells (Fig. 4c). The expression of these miRNAs in CCLP1 was measured by qRT-PCR after transfecting cells with si-linc00473-1 and si-linc00473-2. miR-506 expression was increased and might be one of the potential targets of linc00473 (Fig. 4d, Additional file 2: Fig. S1). And qRT-PCR results showed that the miR-506 expression was significantly lower in 60 CCA tissues (Fig. 4e). Moreover, Pearson correlation analysis revealed a negative association between linc00473 and miR-506 expression in CCA tissues (R = − 0.7582, *p* < 0.0001, Fig. 4f). miR-506 was also less expressed in HCCC-9810 and CCLP1, compared with that in HEBIC (Fig. 4g). Luciferase reporter plasmids with fragments of linc00473 (wild type or mutated) were constructed (Fig. 4h). The results of these assays indicated that the miR-506 mimics significantly decreased the luciferase activity of linc00473-WT instead linc00473-MUT (Fig. 4i, j). In addition, the RIP assay results showed that linc00473 and miR-506 were considerably enriched in Ago2-containing beads, compared with those harboring control IgG (Fig. 4k, l). Taken together, these results suggested that in CCA cells, linc00473 could act as a sponge and reduce miR-506 expression.

### Identification of DDX5 as a direct target gene of miR-506 in CCA cells

**Fig. 5:**
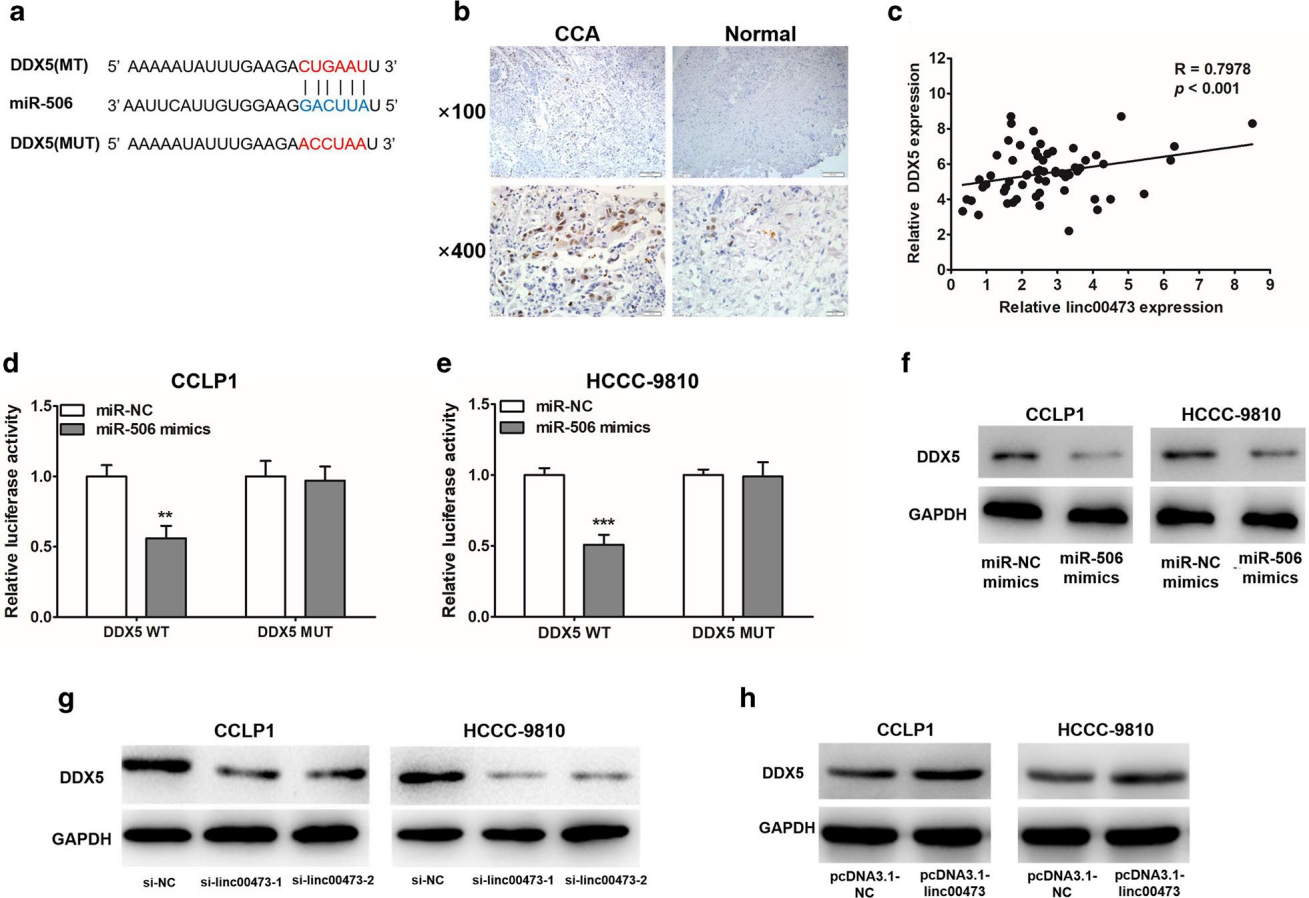
DDX5 was a novel target of miR-506, and linc00473 positively regulated DDX5 through binding with miR-506. **a** The 3′-UTR of DDX-5 harbored miR-506 cognate site. **b** Representative images of DDX5 immunohistochemistry (IHC) staining in normal bile duct tissues and CCA tissues. **c** The expression correlation between linc00473 and DDX5 mRNA in CCA tissues by Pearson’s correlation curve. **d**, **e** CCLP1 and HCCC-9810 were co-transfected with miR-506 mimics and wild-type or mutant DDX-5 3′-UTR for 48 h, the luciferase activity was determined. **f** CCA cells (CCLP1 and HCCC-9810) were transfected with miRNA NC or miR-506 mimics for 48 h, protein level of DDX-5 was determined. **g**, **h** Western blot results indicated that linc00473 depletion significantly decreased DDX5 expression, besides linc00473 overexpression significantly increased DDX5 expression as well. Results shown are the mean ± SD (*p < 0.05, **p < 0.01) of triplicate determination from three independent experiments

The binding site of miR-506 at 3′-UTR of DDX5 mRNA was predicted using starBase (Fig. 5a). The higher protein expression level of DDX5 was further confirmed in specimens by IHC (Fig. 5b). The staining of DDX5 was mostly localized to the nucleus of cancer cells (Fig. 5b). Our group also detected the DDX5 mRNA expression in 60 CCA tissues, and further correlation analysis showed that linc00473 expression levels were positively correlated with those of DDX5 in tissues (Fig. 5c). Dual Luciferase Reporter detection showed miR-506 could bind with 3′-UTR of DDX5 mRNA in CCA cells (Fig. 5d, e). Overexpression miR-506 suppressed the expression of DDX5 protein (Fig. 5f, Additional file 3: Fig. S2a). Meanwhile, the western blot results demonstrated that knockdown of linc00473 led to a decrease of DDX5 (Fig. 5g, Additional file 3: Fig. S2b). Similarly, linc00473 upregulation could increase DDX5 expression in CCLP1 and HCCC-9810 (Fig. 5h, Additional file 3: Fig. S2c). Collectively, these data indicate that DDX5 was a target gene of miR-506, and its expression in CCA cells is coregulated by linc00473 and miR-506.

### miR-506 reversed the carcinogenesis of linc00473

**Fig. 6:**
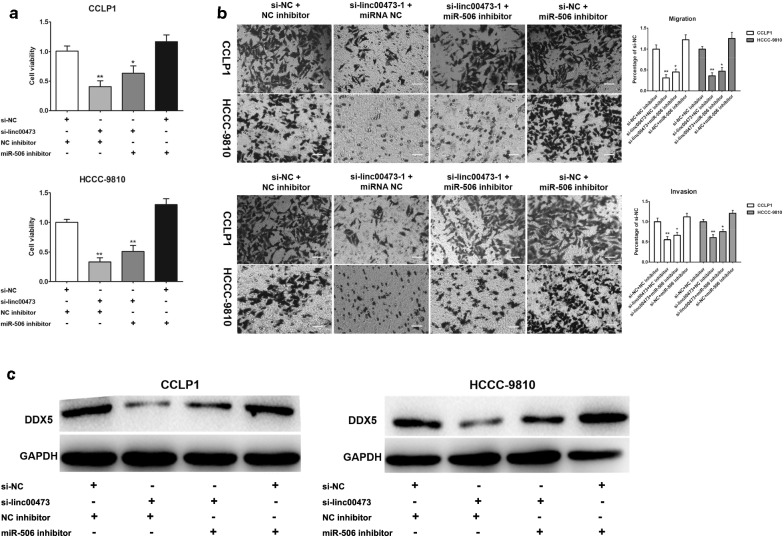
miR-506 reversed the carcinogenesis of linc00473 on CCA cells. **a** CCLP1 and HCCC-9810 cells were transfected with si-linc00473 and miR-506 inhibitor, cell viability of 72 h was detected by CCK8. **b** Cell migration and cell invasion were measured by transwell assays, after CCLP1 and HCCC-9810 cells were transfected with si-linc00473 and miR-506 inhibitor. **c** The expression levels of DDX5 in CCLP1 and HCCC-9810 co-transfected si-linc00473 with miR-506 inhibitor were analyzed by Western blot. The error bars indicate the mean ± SD, and each experiment was repeated at least three times. *p < 0.05, **p < 0.01

Our group continued to investigate the effects of the linc00473/miR-506/DDX5 axis on the proliferation and invasiveness of CCA cells. CCK8 assays revealed that the viability of CCA cells was reduced by si-linc00473 transfection and was increased by miR-506 inhibitor transfection (Fig. 6a). Compared with that in the control group, the knockdown of linc00473 decreased the number of migratory and invasive cells among CCA cells, but this effect was reversed by co-transfection of the miR-506 inhibitor (Fig. 6b). As expected, miR-506 inhibitor could increase DDX5 expression in CCA cells transfected with si-linc00473 (Fig. 6c, Additional file 4: Fig. S3). In a summary, miR-506 could reverse the carcinogenesis of linc00473 in CCA.

### Ectopic expression of linc00473 promoted CCA growth in vivo

**Fig. 7:**
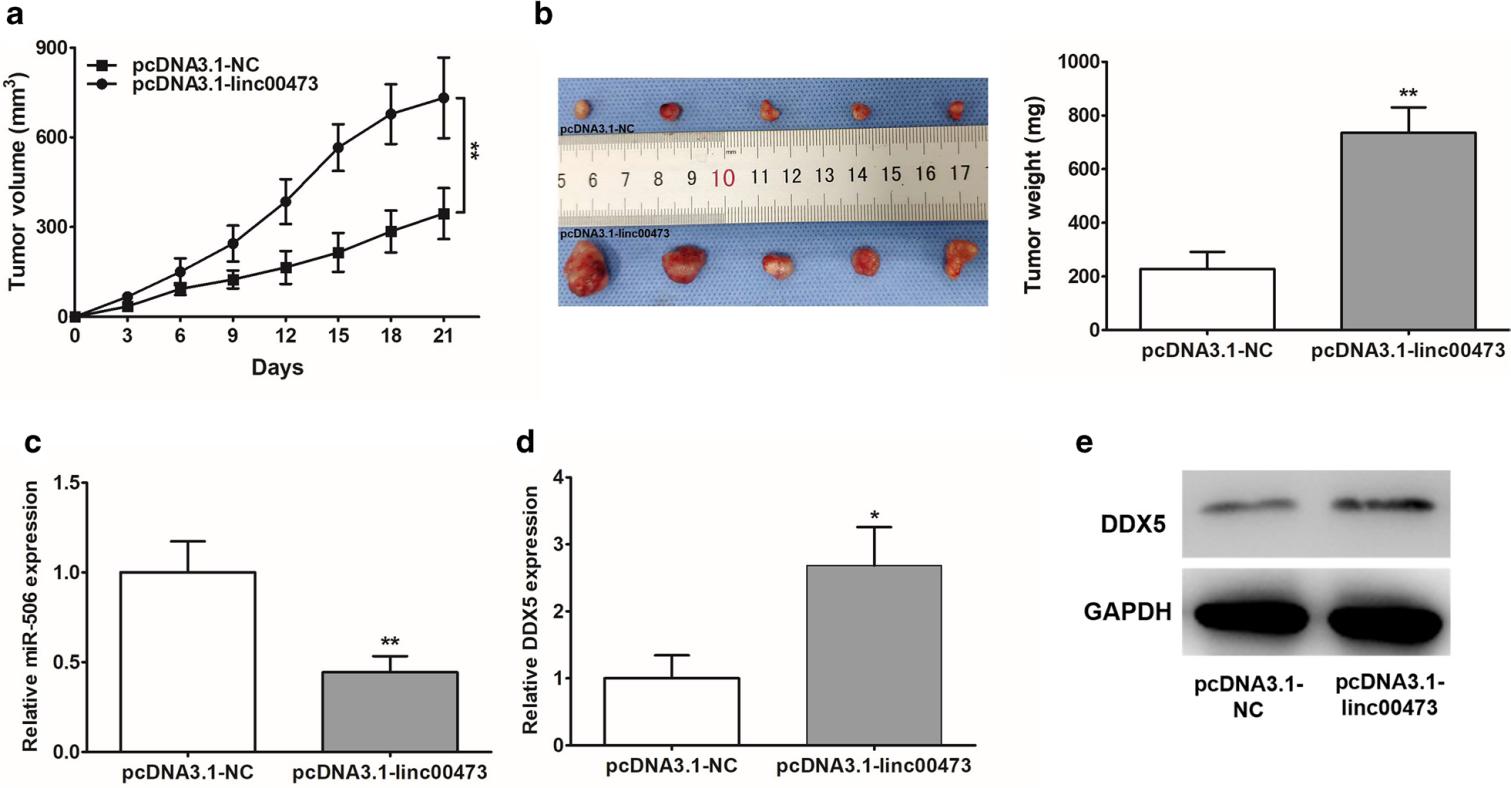
Linc00473 up-regulation increased tumor growth. **a**, **b** CCLP1 cells transfected with pcDNA3.1-linc00473 or pcDNA3.1-NC were separately injected subcutaneously into nude mice. On the 21st day following the injection, the tumors were removed after euthanasia. The tumor volumes were calculated every 3 days and the tumor weights were measured after excision. The results confirmed that linc00473 overexpression increased both the tumor volumes and the tumor weights. **c**, **d** The expression of miR-506 and DDX5 mRNA was detected by qRT-PCR. **e** Western blot results indicated that DDX5 expression was significantly increased in pcDNA3.1-linc00473 group. The error bars indicate the mean ± SD, and each experiment was repeated at least three times. *p < 0.05, **p < 0.01

To ascertain our in vitro findings, we established an in vivo xenograft model in nude mice. pcDNA3.1-linc00473 was introduced into CCLP1 cells, which were then inoculated into the nude mice. Tumors were allowed to form and grow for 21 days, and the tumor size and weight were measured at regular intervals during this period. The results showed that the average volume and weight of tumors were significantly higher in pcDNA3.1-linc00473 groups (Fig. 7a, b). qRT-PCR analysis was conducted to measure miR-506 and DDX5 mRNA level, miR-506 expression was declined in the pcDNA3.1-linc00473 group when compared with pcDNA3.1-NC group (Fig. 7c), and the DDX5 mRNA and protein expression were also increased when linc00473 was up-regulated (Fig. 7d, e).

## Discussion

CCA remains one of the most aggressive malignant disease with unsatisfactory curative effects in clinical practice. Although the survival of CCA patients has significantly progressed over the past decades, the prognosis of patients remains poor, with 5-year overall survival rates of 10–15%. Additionally, recent studies have indicated that the abnormally expressed cancer-related gene was considered to be key factors in the CCA development, and lncRNAs have been demonstrated to emerge as an important regulator of CCA. For instance, lncRNA-MEG3 inhibited cell proliferation and invasion by modulating Bmi1/RNF2 in CCA. LncRNA NNT-AS1 promoted the expression of BCL9 by targeting miR-485 and regulated cell growth and invasion in CCA [17]. However, the problem of occurrence and progression for CCA remains to be solved.

It is necessary to explore the biological process of CCA and help us find out potential therapeutic targets. Our group examined the expression and biological functions of linc00473 in CCA. Linc00473 was highly expressed in CCA, and higher linc00473 expression had a close correlation with shorter overall survival time. Then our group detected in vitro and in vivo assays to explore the function of linc00473 in CCA progression. The results suggested that linc00473 deletion issued significant inhibition of cell proliferation, migration and invasion, and linc00473 up-regulation promoted tumor growth in nude mice xenografts. To further explore the mechanism by which linc00473 regulated CCA cell proliferation and invasion, we performed bioinformatic analysis and in vitro experimental studies and found that lin00473 could sponge miR-506, which play a role in cancer suppression in various cancers [18, 19]. In short, these data suggested that linc00473 could have an oncogenic effect on CCA by negatively regulating miR-506.

Then, we identified that miR-506 regulated gene expression by binding to the 3′-UTR of DDX5. DDX5 was a member of a family of highly conserved proteins involved in gene-expression regulation and ATP-dependent RNA helicase activities. DDX5 was well-known regulators of various transcription factors, of alternative splicing and of miRNA expression. DDX5 was closely related to multiple cell processes, including transcription regulation [20], promoting mRNA processing [21], promoting microRNA processing [22] and ribosome biogenesis [23]. DDX5 also played important roles in promoting cancer cell proliferation and metastasis [24]. Published studies have shown that DDX5 enhances glioma cells invasion by negatively regulating DUSP5 [25]. Xue et al. [26] also found that DDX5 increased hepatocellular carcinoma cells growth through activating Akt signaling pathway. Moreover, our results revealed that miR-506 inhibited DDX5 expression by directly binding to the 3′UTR region of DDX5 mRNA. DDX5 has been reported to be correlated with Wnt/β-catenin signaling pathway, DDX5 could affect β-catenin by protecting β-catenin from degradation via dissociation from the cytoplasmic APC/axin/GSK-3β complex in the cytoplasm [27]. In the current study, we identified that linc00473 knockdown inhibited DDX5 expression, similarly, overexpression of linc00473 promoted DDX5 expression. Moreover, linc00473 appeared to positively regulate DDX5 expression. Though cell experiments, we found that linc00473 regulated DDX5 expression by sponging miR-506 in CCA.

## Conclusion

In our study, linc00473 promoted DDX5 expression by sponging miR-506, thus promoting CCA cell proliferation and invasion in vitro and CCA tumor growth in vivo. Moreover, linc00473 was found to be up-regulated in CCA tissue samples and inversely correlated with miR-506 levels but positively correlate with DDX5 expression. Together with further research, these findings indicated that linc00473 might be a potential therapeutic target for CCA treatment.

## Supplementary information

**Additional file 1.** Primers used in real-time PCR analysis.

**Additional file 2: Fig. S1.** HCCC-9810 cells were transfected with si-NC, si-linc00473-1 or si-linc00473-2 for 24 h, expression of miRNAs was detected.

**Additional file 3: Fig. S2. a** Western blot assay examined DDX5 protein level after transfection with miRNA NC or miR-506 mimics. **b** Western blot detected DDX5 expression by linc00473 depletion. **c** Western blot analysis of DDX5 expressions under linc00473 overexpression.

**Additional file 4: Fig. S3.** The DDX5 expression levels in CCLP1 and HCCC-9810 co-transfected si-linc00473 with miR-506 inhibitor were analyzed by Western blot assay.

## Data Availability

The original data is stored in the first author and the correspondent author, and all experimental raw data can be obtained from any one of them if necessary.

## Abbreviations

CCA: Cholangiocarcinoma
ceRNA: Competitive endogenous RNA
CCK-8: Cell counting kit-8
DDX5: The DEAD box protein 5
HIBEC: Human intrahepatic biliary epithelial cells
IHC: Immunohistochemistry
lnRNA: Long non-coding RNA
WT: Wild-type
MUT: Mutated type
RIP: RNA immunoprecipitation
TCGA: The Cancer Genome Atlas

## References

[1] Razumilava N, Gores GJ (2014). Cholangiocarcinoma. Lancet (London, England).

[2] Rizvi S, Khan SA, Hallemeier CL, Kelley RK, Gores GJ (2018). Cholangiocarcinoma—evolving concepts and therapeutic strategies. Nat Rev Clin Oncol.

[3] Massironi S, Pilla L, Elvevi A, Longarini R, Rossi RE, Bidoli P, Invernizzi P (2020). New and emerging systemic therapeutic options for advanced cholangiocarcinoma. Cells.

[4] Bhan A, Soleimani M, Mandal SS (2017). Long noncoding RNA and cancer: a new paradigm. Cancer Res.

[5] Chi Y, Wang D, Wang J, Yu W, Yang J (2019). Long non-coding RNA in the pathogenesis of cancers. Cells.

[6] Kolenda T, Guglas K, Kopczyńska M, Teresiak A, Bliźniak R, Mackiewicz A, Lamperska K, Mackiewicz J (2019). Oncogenic role of ZFAS1 lncRNA in head and neck squamous cell carcinomas. Cells.

[7] Wang Y, Chen W, Lian J, Zhang H, Yu B, Zhang M, Wei F, Wu J, Jiang J, Jia Y (2020). The lncRNA PVT1 regulates nasopharyngeal carcinoma cell proliferation via activating the KAT2A acetyltransferase and stabilizing HIF-1α. Cell Death Differ.

[8] Wang N, Zhang C, Wang W, Liu J, Yu Y, Li Y, Zhang M, Ge X, Li Q, Miao L (2019). Long noncoding RNA DANCR regulates proliferation and migration by epigenetically silencing FBP1 in tumorigenesis of cholangiocarcinoma. Cell Death Dis.

[9] Chen Z, Li J-L, Lin S, Cao C, Gimbrone NT, Yang R, Fu DA, Carper MB, Haura EB, Schabath MB (2016). cAMP/CREB-regulated LINC00473 marks LKB1-inactivated lung cancer and mediates tumor growth. J Clin Invest.

[10] Zhang W, Song Y (2018). LINC00473 predicts poor prognosis and regulates cell migration and invasion in gastric cancer. Biomed Pharma.

[11] Shi C, Yang Y, Yu J, Meng F, Zhang T, Gao Y (2017). The long noncoding RNA LINC00473, a target of microRNA 34a, promotes tumorigenesis by inhibiting ILF2 degradation in cervical cancer. Am J Cancer Res.

[12] Mo J, Li B, Zhou Y, Xu Y, Jiang H, Cheng X, Wu X, Zhang Y (2019). LINC00473 promotes hepatocellular carcinoma progression via acting as a ceRNA for microRNA-195 and increasing HMGA2 expression. Biomed Pharma.

[13] Li J, Jiang X, Li C, Liu Y, Kang P, Zhong X, Cui Y (2019). LncRNA-MEG3 inhibits cell proliferation and invasion by modulating Bmi1/RNF2 in cholangiocarcinoma. J Cell Physiol.

[14] Li X, Du N, Zhang Q, Li J, Chen X, Liu X, Hu Y, Qin W, Shen N, Xu C (2014). MicroRNA-30d regulates cardiomyocyte pyroptosis by directly targeting foxo3a in diabetic cardiomyopathy. Cell Death Dis.

[15] Zhang Y, Li X, Zhang Q, Li J, Ju J, Du N, Liu X, Chen X, Cheng F, Yang L (2014). Berberine hydrochloride prevents postsurgery intestinal adhesion and inflammation in rats. J Pharmacol Exp Ther.

[16] Leng K, Xu Y, Kang P, Qin W, Cai H, Wang H, Ji D, Jiang X, Li J, Li Z (2019). Akirin2 is modulated by miR-490-3p and facilitates angiogenesis in cholangiocarcinoma through the IL-6/STAT3/VEGFA signaling pathway. Cell Death Dis.

[17] Huang L, Jiang X, Kang P, Wang Z, Leng K, Ji D, Xu Y, Wang H, Cui Y (2019). Long non-coding RNA NNT-AS1 functions as an oncogenic gene through modulating miR-485/BCL9 in cholangiocarcinoma. Cancer Manag Res.

[18] Liang T-S, Zheng Y-J, Wang J, Zhao J-Y, Yang D-K, Liu Z-S (2019). MicroRNA-506 inhibits tumor growth and metastasis in nasopharyngeal carcinoma through the inactivation of the Wnt/β-catenin signaling pathway by down-regulating LHX2. J Exp Clin Cancer Res.

[19] Yong W, Yu D, Jun Z, Yachen D, Weiwei W, Midie X, Xingzhu J, Xiaohua W (2018). Long noncoding RNA NEAT1, regulated by LIN28B, promotes cell proliferation and migration through sponging miR-506 in high-grade serous ovarian cancer. Cell Death Dis.

[20] Engreitz JM, Haines JE, Perez EM, Munson G, Chen J, Kane M, McDonel PE, Guttman M, Lander ES (2016). Local regulation of gene expression by lncRNA promoters, transcription and splicing. Nature.

[21] Ma WK, Paudel BP, Xing Z, Sabath IG, Rueda D, Tran EJ (2016). Recruitment, duplex unwinding and protein-mediated inhibition of the dead-box RNA helicase Dbp2 at actively transcribed chromatin. J Mol Biol.

[22] Li H, Lai P, Jia J, Song Y, Xia Q, Huang K, He N, Ping W, Chen J, Yang Z (2017). RNA Helicase DDX5 inhibits reprogramming to pluripotency by miRNA-based repression of RYBP and its PRC1-dependent and -independent functions. Cell Stem Cell.

[23] Tedeschi FA, Cloutier SC, Tran EJ, Jankowsky E (2018). The DEAD-box protein Dbp2p is linked to noncoding RNAs, the helicase Sen1p, and R-loops. RNA (New York, NY).

[24] Huang W, Thomas B, Flynn RA, Gavzy SJ, Wu L, Kim SV, Hall JA, Miraldi ER, Ng CP, Rigo F (2015). DDX5 and its associated lncRNA Rmrp modulate TH17 cell effector functions. Nature.

[25] Wang R, Bao H-B, Du W-Z, Chen X-F, Liu H-L, Han D-Y, Wang L-G, Wu J-N, Wang C-L, Yang M-C (2019). P68 RNA helicase promotes invasion of glioma cells through negatively regulating DUSP5. Cancer Sci.

[26] Xue Y, Jia X, Li L, Dong X, Ling J, Yuan J, Li Q (2018). DDX5 promotes hepatocellular carcinoma tumorigenesis via Akt signaling pathway. Biochem Biophys Res Commun.

[27] Shin S, Rossow KL, Grande JP, Janknecht R (2007). Involvement of RNA helicases p68 and p72 in colon cancer. Cancer Res.

