# Supplementary material for: Linc00473 potentiates cholangiocarcinoma progression by modulation of DDX5 expression via miR-506 regulation
Source: Cancer Cell Int. 2020 Jul 18;20:324. doi: 10.1186/s12935-020-01415-4 (PMC7368746; doi:10.1186/s12935-020-01415-4)
Supplement: Supplementary file 1 — Additional file 1. Primers used in real-time PCR analysis. [file 12935_2020_1415_MOESM1_ESM.doc]

**Linc00473 potentiates** **cholangiocarcinoma progression by modulation of DDX5 expression via miR-506 regulation**

Lining Huang, Xingming Jiang, Zhenglong Li, Jinglin Li, Xuan Lin, Zengtao Hu, Yunfu Cui*

Department of General Surgery, The 2nd Affiliated Hospital of Harbin Medical University, Harbin 150086, P.R. China

*Corresponding Author

Yunfu Cui

Department of General Surgery, The 2nd Affiliated Hospital of Harbin Medical University, 246 Xuefu-ro, Harbin 150086, P.R. China

E-mail: yfcui777@hotmail.com

ORCID

Lining Huang 0000-0003-1177-4530

**Additional file 1 Primers used in real-time PCR analysis**

| Gene | Primer sequence (5’-3’) | Species |
| --- | --- | --- |
| miR-15 | Forward: GCGCAGGCCATATTGTGCT  Reverse: AGTGCAGGGTCCGAGGTATT | Human |
| miR-130 | Forward: CGCGGCTCTTTTCACATTGT  Reverse: AGTGCAGGGTCCGAGGTATT | Human |
| miR-133 | Forward: GCGTTTGGTCCCCTTCAAC | Human |
|  | Reverse: AGTGCAGGGTCCGAGGTATT |  |
| miR-139 | Forward: CGCGTCTACAGTGCACGTGTC | Human |
|  | Reverse: AGTGCAGGGTCCGAGGTATT |  |
| miR-142 | Forward: GCGCGTGTAGTGTTTCCTACTT | Human |
|  | Reverse: AGTGCAGGGTCCGAGGTATT |  |
| miR-195 | Forward: CGCCAATATTGGCTGTGC | Human |
|  | Reverse: AGTGCAGGGTCCGAGGTATT |  |
| miR-431 | Forward: CGCAGGTCGTCTTGCAGG | Human |
|  | Reverse: AGTGCAGGGTCCGAGGTATT |  |
| miR-545 | Forward: GCGCGTCAGCAAACATTTATT | Human |
|  | Reverse: AGTGCAGGGTCCGAGGTATT |  |
| miR-506 | Forward: CGCGTAAGGCACCCTTCTG | Human |
|  | Reverse: AGTGCAGGGTCCGAGGTATT |  |
| U6 | Forward: AGAGCCTGTGGTGTCCG | Human |
|  | Reverse: CATCTTCAAAGCACTTCCCT |  |
| GAPDH | Forward: GGGAGCCAAAAGGGTCAT | Human |
|  | Reverse: GAGTCCTTCCACGATACCAA |  |
| linc00473 | Forward: GAGTCGTTCCTGAGAGCACA | Human |
|  | Reverse: CTCCTTCCCTCCTTTCCATC |  |
