# Supplementary figures and images for: Linc00473 potentiates cholangiocarcinoma progression by modulation of DDX5 expression via miR-506 regulation
Source: Cancer Cell Int. 2020 Jul 18;20:324. doi: 10.1186/s12935-020-01415-4 (PMC7368746; doi:10.1186/s12935-020-01415-4)

### HCCC-9810

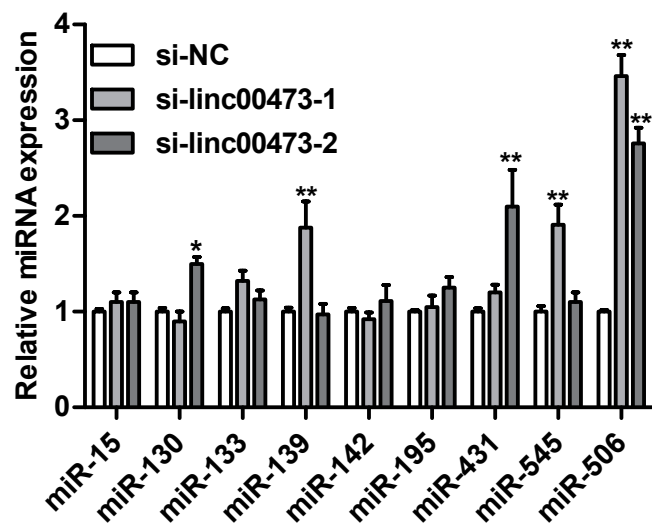

Supplement: Supplementary file 2 — Additional file 2: Fig. S1. HCCC-9810 cells were transfected with si-NC, si-linc00473-1 or si-linc00473-2 for 24 h, expression of miRNAs was detected. [file 12935_2020_1415_MOESM2_ESM.pdf]

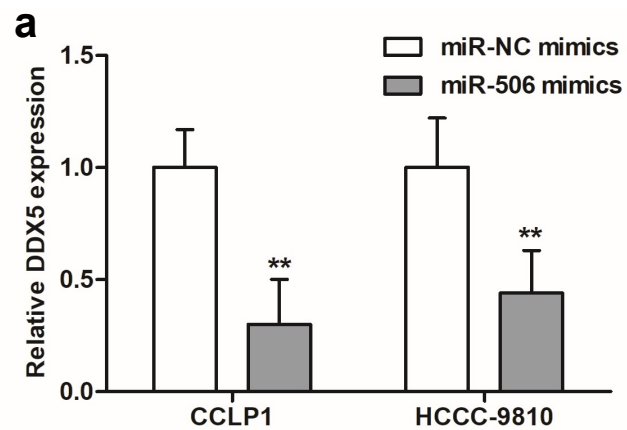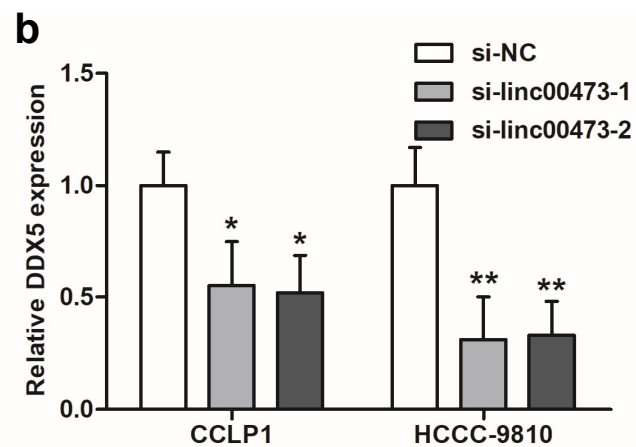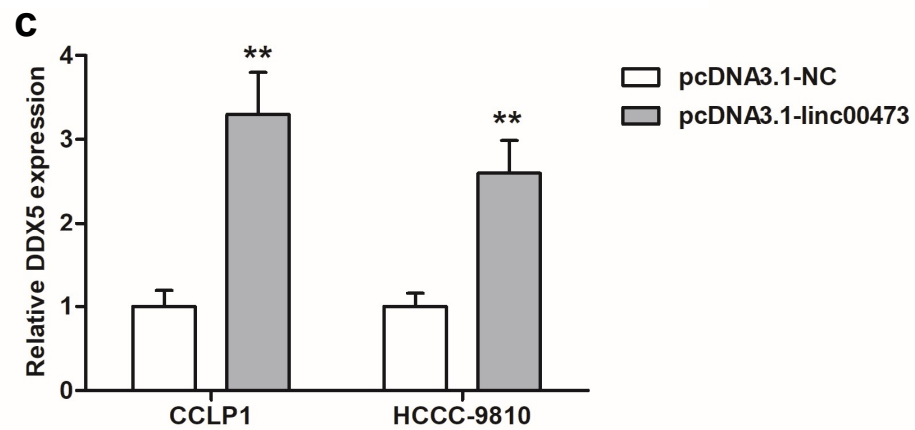

Supplement: Supplementary file 3 — Additional file 3: Fig. S2. a Western blot assay examined DDX5 protein level after transfection with miRNA NC or miR-506 mimics. b Western blot detected DDX5 expression by linc00473 depletion. c Western blot analysis of DDX5 expressions under linc00473 overexpression. [file 12935_2020_1415_MOESM3_ESM.pdf]

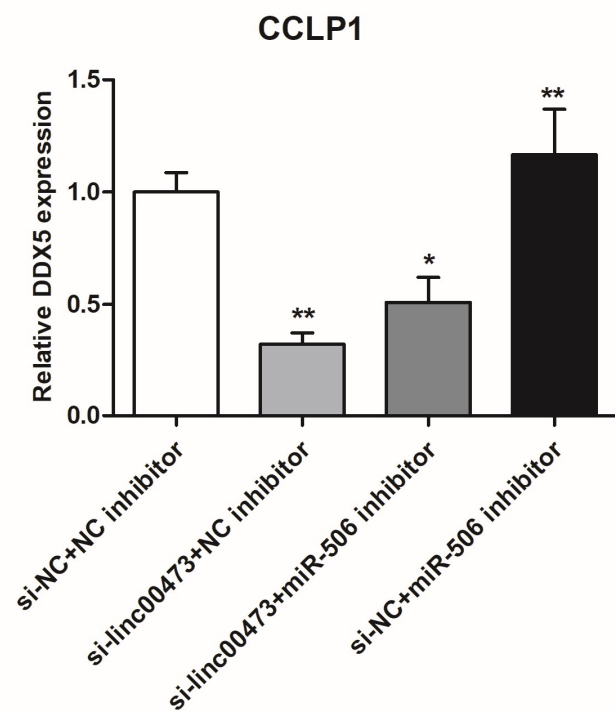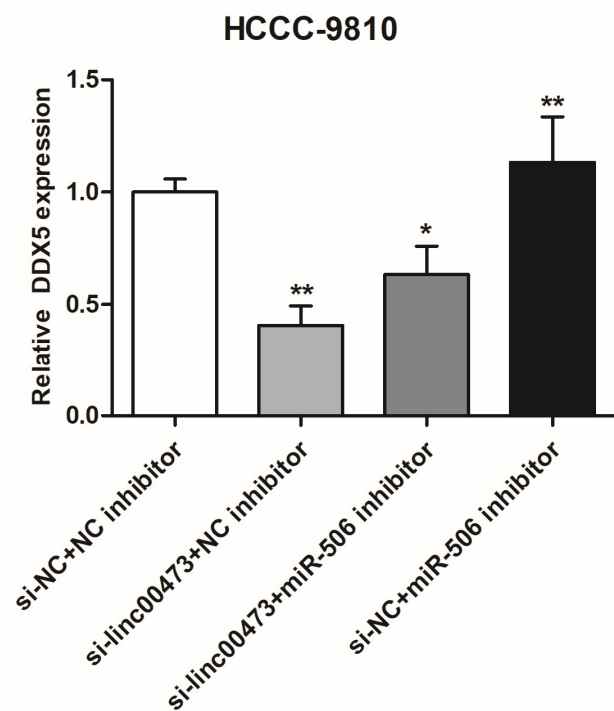

Supplement: Supplementary file 4 — Additional file 4: Fig. S3. The DDX5 expression levels in CCLP1 and HCCC-9810 co-transfected si-linc00473 with miR-506 inhibitor were analyzed by Western blot assay. [file 12935_2020_1415_MOESM4_ESM.pdf]
